# Desynchronization of diurnal rhythms in bipolar disorder and borderline personality disorder

**DOI:** 10.1038/s41398-018-0125-7

**Published:** 2018-04-12

**Authors:** Oliver Carr, Kate E. A. Saunders, Amy C. Bilderbeck, Athanasios Tsanas, Niclas Palmius, John R. Geddes, Russell Foster, Maarten De Vos, Guy M. Goodwin

**Affiliations:** 1 Institute of Biomedical Engineering, Department of Engineering Science, University of Oxford, Oxford, OX3 7DQ UK; 2 University Department of Psychiatry, University of Oxford, Oxford, OX3 7JX UK; 3 Oxford Health NHS Foundation Trust, University of Oxford, Oxford, OX3 7JX UK; 4 Usher Institute of Population Health Sciences and Informatics, Medical School, University of Edinburgh, Edinburgh, EH16 4UX UK; 5 Oxford Centre for Industrial and Applied Mathematics, Mathematical Institute, University of Oxford, Oxford, OX26GG UK; 6 Sleep and Circadian Neuroscience Institute, Nuffield Department of Clinical Neurosciences, University of Oxford, Oxford, OX3 9DU UK

## Abstract

It has long been proposed that diurnal rhythms are disturbed in bipolar disorder (BD). Such changes are obvious in episodes of mania or depression. However, detailed study of patients between episodes has been rare and comparison with other psychiatric disorders rarer still. Our hypothesis was that evidence for desynchronization of diurnal rhythms would be evident in BD and that we could test the specificity of any effect by studying borderline personality disorder (BPD). Individuals with BD (*n* = 36), BPD (*n* = 22) and healthy volunteers (HC, *n* = 25) wore a portable heart rate and actigraphy device and used a smart-phone to record self-assessed mood scores 10 times per day for 1 week. Average diurnal patterns of heart rate (HR), activity and sleep were compared within and across groups. Desynchronization in the phase of diurnal rhythms of HR compared with activity were found in BPD (+3 h) and BD (+1 h), but not in HC. A clear diurnal pattern for positive mood was found in all subject groups. The coherence between negative and irritable mood and HR showed a four-cycle per day component in BD and BPD, which was not present in HC. The findings highlight marked de-synchronisation of measured diurnal function in both BD but particularly BPD and suggest an increased association with negative and irritable mood at ultradian frequencies. These findings enhance our understanding of the underlying physiological changes associated with BPD and BD, and suggest objective markers for monitoring and potential treatment targets. Improved mood stabilisation is a translational objective for management of both patient groups.

## Introduction

Human biology is circadian. The central oscillator or clock in the suprachiasmatic nucleus of the brain has a harmonic cycle of about 24 h and a sleep homeostat overlays the clock. The sleep/wake cycle is the most obvious expression of this physiology. Sleep is commonly disturbed in psychiatric disorder. In the case of bipolar disorder (BD), sleep deprivation leads to mood elevation and mania^1^. This striking linkage is often used to claim that circadian rhythms are disturbed in mood disorders^2,3^. However, a sleep effect does not automatically imply involvement of the circadian clock although sleep abnormalities appear to be associated with some other diurnal measures in bipolar disorder^4^. The relationship between mood and diurnal function is of particular interest when patients are apparently well, because abnormal sensitivity to different aspects of sleep or the circadian cycle might explain why patients with BD show persistent mood instability in euthymia or have the potential to decompensate into mania or depression^5^. Accordingly, it is when patients are well and between episodes that interventions to stabilise mood must be developed and deployed.

We have previously suggested that mood instability may be an important target for testing the potential efficacy of possible mood stabilising treatments^6^. However, experimental investigation of mood instability has been very limited and largely confined to the study of symptom patterns. A link with possible disturbances of diurnal physiology has not hitherto been demonstrated. However, diurnal changes in daytime activity have been found to differentiate BD participants and controls^7^. Wearable devices which record quantitative behavioural and physiological signals that enable collection of passive data streams. Such devices offer the potential to analyse objective correlates of mood symptoms.

In the present study patients with either BD or borderline personality (BPD), together with healthy volunteers (HC), were studied in an observational protocol, which yielded intensive data over several days. As indicated, BD has long been of interest from a circadian perspective. BPD resembles BD in that patients experience mood instability, but this variability of mood is greater^6^. Descriptions of sleep difficulties in BPD refer quite non-specifically to insomnia^8^. The BPD group were a relevant control group because the diagnoses are sometimes confused even though heritability and the clinical course are different from BD. Our hypothesis was that disruption of the synchrony of diurnal physiology would be disturbed in BD and we would test whether the effect was specific to the BD phenotype, as is often claimed or assumed, by comparison with BPD. Our methodology was to observe the diurnal variation in total activity and relate this to vertical acceleration (an approximation of sleep per se) and to heart rate (HR). We have also looked at diurnal or ultradian correlations between these objective measures of physiology and subjective mood sampled intensively up to ten times daily over the same time interval.

Patients were studied in the mood state that was most normal for them. For BD, they were not syndromally depressed or hypomanic. However, they were symptomatic as the majority of BD patients are most of the time. BPD patients were not in a crisis but they were symptomatic, as they are almost all the time. These states are of particular interest because the challenge for the long term is the risk of relapse (BD) or crisis (BPD) and the translational need is to innovate new mood stabilising interventions.

## Methods

A total of 129 participants were recruited (54 BD, 31 BPD and 44 healthy controls (HC)) as part of the Automated Monitoring of Symptom Severity (AMoSS) study at the University of Oxford^9^, with participant information summarised in the supplementary information. The study was approved by the NRES Committee East of England—Norfolk (13/EE/0288). All participants gave written informed consent. The AMoSS study collected behavioural data from participants^9^, in addition to self-reported mood scores using a smartphone app (Mood Zoom or MZ), and self-reported clinically validated questionnaires to capture psychiatric symptoms^6,10^. Participants diagnosed with BD or BPD and healthy controls were recruited for the study through previous studies, local advertising and word-of-mouth. All the participants were screened by an experienced psychiatrist (KEAS) using the structured clinical interview for DSM IV and the BPD module of the international personality disorders examination. The HC participants were excluded if they had any history of depression or screened positive for an axis 1 disorder or BPD. They agreed initially for 3 month period of recording, with the option of continuing participating in the study and providing mood recordings beyond the first 3 months. Data collection started in March 2014 and the data analysed here were collected up until February 2016. The self reported MZ mood ratings and the clinical ratings have been described already in relation to their variability across the study groups^6,9,10^, but not in relation to the diurnal pattern to be illustrated here.

For 1 week during the study, the participants underwent ‘high intensity’ monitoring, during which multiple signals were recorded from a Proteus patch (www.proteus.com), stuck to the torso of participants. The patch was not removed during the whole ‘high intensity’ week and recorded HR and acceleration in three directions at one sample per minute. The patch recorded HR and acceleration in three directions, with ‘estimated sleep period’ measured from vertical acceleration which determines when participants are lying down (see ref. ^11^).

Mood data were collected through the MZ smart-phone application, which prompted participants to provide their mood 10 times evenly spaced throughout each day. The prompts typically started at 1000 hours and finished at 2000 hours, thus on average mood characteristics were self-reported every hour. Participants rated six mood items in the smart-phone application: anxious, elated, sad, angry, irritable and energetic on a seven point Likert scale from zero, corresponding to ‘not at all’, to six, corresponding to ‘very much’. For further details on MZ see Tsanas et al.^6^.

The HR data obtained from the Proteus patch gave an average over 5 min intervals; it did not permit measurement of conventional beat-to-beat HR variability. However, the data allowed for long term analysis of diurnal patterns in physiological and behavioural measures. The acceleration signals are recorded in vertical, horizontal and forward directions, with a sampling frequency of 0.017 Hz (once per minute).

**Fig. 1:**
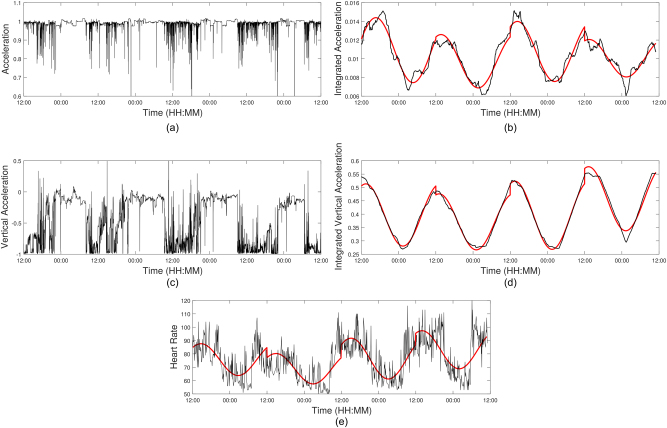
An example of data recorded from the Proteus patch for one participant. **a** total acceleration, **b** integrated total acceleration with daily sinusoid fits, **c** vertical acceleration, **d** integrated vertical acceleration with daily sinusoid fits, and **e** HR with daily sinusoid fits

The data obtained from the Proteus patch, which is shown in Fig. 1, allows broad band analysis of both physiological and behavioural measures of diurnal rhythm. However, in practice, viable recordings from each participant were of variable durations, ranging from 1 day to over 8 days. For time domain analysis of average diurnal rhythms, data were processed from: 38 BD participants (13 male and 25 female with a mean age of 39.7 ± 12.6 years and a mean BMI of 27.2 ± 5.1 kg/m^2^), 22 BPD participants (2 male and 20 female with a mean age of 34.4 ± 10.9 years and a mean BMI of 27.5 ± 5.9 kg/m^2^), and 27 HC participants (6 male and 21 female with a mean age of 40.9 ± 13.9 years and a mean BMI of 24.8 ± 4.0 kg/m^2^). Data from other participants was not used only if their acceleration and HR signals were missing or of poor quality. This was due to a number of reasons: the devices not recording any data, the patch falling off or being removed before 4 days of recording and devices recording noisy data with large numbers or artefacts. Details on the non-participants are provided in Table 1.

**Table 1:** Median and interquartile range of the average phases, MESORs and amplitudes of the daily sinusoid fits for each of the participant groups, the difference between phases of each modality, and statistical significance pairwise comparisons across the three groups (BD *n* = 36, BPD *n* = 22 and HC *n* = 25) using the Wilcoxon statistical hypothesis test

Within group phase differences tested against zero mean						
	BD	BPD	HC	BD (FDR)	BPD (FDR)	HC (FDR)
HR-ACT (mean ± std)	0.72 ± 4.17	3.11 ± 3.08	0.46 ± 4.06	**0.017**	**<0.001**	0.246
HR-SLP (mean ± std)	0.56 ± 3.41	0.99 ± 1.66	−0.83 ± 2.11	**0.006**	**0.006**	0.051
ACT-SLP (mean ± std)	−0.16 ± 2.90	−2.12 ± 3.21	−1.29 ± 2.87	0.180	**0.005**	**0.004**
**Group differences in phase, MESOR and amplitude**						
	**BD**	**BPD**	**HC**	**BD vs BPD (FDR)**	**BD vs HC (FDR)**	**BPD vs HC (FDR)**
Activity						
Phase (mean ± std)	14.09 ± 2.83	12.34 ± 3.57	12.82 ± 3.31	0.253	0.410	0.403
MESOR (mean ± std)	0.014 ± 0.004	0.013 ± 0.003	0.014 ± 0.004	0.276	0.418	0.418
Amplitude (mean ± std)	0.0033 ± 0.0021	0.0026 ± 0.0014	0.0027 ± 0.0013	0.294	0.401	0.380
Sleep						
Phase (mean ± std)	14.25 ± 1.68	14.46 ± 2.07	14.12 ± 1.31	0.398	0.483	0.391
MESOR (mean ± std)	0.35 ± 0.08	0.34 ± 0.07	0.36 ± 0.07	0.477	0.411	0.385
Amplitude (mean ± std)	0.11 ± 0.02	0.11 ± 0.03	0.11 ± 0.02	0.325	0.444	0.276
Heart rate						
Phase (mean ± std)	14.81 ± 3.21	15.45 ± 2.07	13.28 ± 2.37	0.384	0.086	0.051
MESOR (mean ± std)	75.24 ± 7.08	77.65 ± 5.69	74.48 ± 2.62	0.251	0.425	0.275
Amplitude (mean ± std)	8.10 ± 2.54	8.67 ± 3.45	7.41 ± 2.62	0.369	0.300	0.289

To test phase differences against zero mean within groups, a Mann–Whitney test was used at 5% significance level. Correction for multiple testing was performed using the false discovery rate (FDR)

Statistically significant differences at the *p* = 0.05 level appear in bold. avPhase, avMESOR and avAmplitude denote the mean of the phases, MESORs and amplitudes of the daily sinusoid fits. The units of all phase measures are shown in hours

For frequency domain analysis, only participants who had 4 or more days of recording provided a good representation of diurnal variability. For these participants, the first 4 days of the recording were selected to standardise the analysis. Thus, we processed data from: 18 BD participants (4 male and 14 female with a mean age of 40.9 ± 11.4 years and a mean BMI of 26.1 ± 3.9 kg/m^2^), 14 BPD participants (2 male and 12 female with a mean age of 34.3 ± 10.2 years and a mean BMI of 25.9 ± 5.1 kg/m^2^) and 20 HC participants (4 male and 16 female with a mean age of 43.4 ± 14.6 years and a mean BMI of 24.6 ± 4.2 kg/m^2^).

### Mood zoom data

Principal component analysis was applied to the pooled MZ data from all participants in the study across all groups, with the first three components used to quantify the mood of participants^6^. The first principal component is a measure of negative mood, the second is a measure of positive mood and the third principal component is a measure of irritability^10^.

The average diurnal rhythms of mood were found through linear interpolation of the mood entries to an evenly spaced sample frequency of 0.017 Hz (or one sample per minute) and the average was taken across all available 24 h periods.

### Extracting diurnal rhythms

Figure 1 shows the behavioural and physiological data from a randomly chosen single subject (total acceleration, vertical acceleration and HR) to demonstrate diurnal rhythms. Acceleration data were integrated to give a smooth measure of movement (the integration was performed by removing the mean and then integrating an overlapping window of 6 h which is shifted by one data point along the entire signal). There is an obvious first harmonic at around 1 cycle/24 h, corresponding to the diurnal period.

The HR data were pre-processed to remove artefacts in the signal. The value of HR at each recorded instance was compared to the value recorded at the preceding time instance: if the value was over 150% or under 50% of the previous value the value at the current time was set to the mean of the preceding and succeeding values, with a mean of 0.9% ± 1.1% of the data being adjusted for each participant. An indicative example of the recorded data is presented in Fig. 1. The HR signals again exhibit natural sinusoidal behaviour as can be seen in Fig. 1e, like the integrated acceleration signals in Fig. 1b, d.

Diurnal rhythms were quantified by fitting a sine wave to a 24 h signal by quantifying the phase, average value and amplitude, known as the Cosinor method^12–14^. Here, this analysis has been extended to longer term signals over a number of days through fitting of sine waves to each separate day of the signal for integrated total acceleration, integrated vertical acceleration and HR, before finding the average of the daily sinusoids, shown in Fig. 1b, d, e (see ref. ^11^). Detailed methods of fitting sinusoids are shown in the supplementary materials.

### Coherence analysis

The magnitude-squared coherence was calculated for the nine pairs of acceleration and HR recordings with the three principal components of mood. Coherence is used to determine the linear relationship between two signals through their frequency spectrum, or finding the correlation in the frequency domain. The magnitude squared coherence is calculated as:

1 $$C_{xy}\left( f \right) = \frac{{|G_{xy}\left( f \right)|^2}}{{G_{xx}(f)G_{yy}(f)}}$$

where *G*_*xy*_*(f)* represents the cross-spectral density between signals *x* and *y*, and *G*_*xx*_*(f)* and *G*_*yy*_*(f)* represent the autospectral density of signals *x* and *y*, respectively.

A coherence value of one indicates an ideal linear system at a certain frequency, whereas zero indicates the two signals are not linearly related. A value between zero and one indicates the fraction of the output signal which is produced by the input signal at each given frequency. The coherence analysis was only performed on participants with over 4 days of recorded data in order to express the relationship between the diurnal variations in the signal. Coherence was calculated on the entire length of the raw signals, with a minimum of 1 day up to 8 days, where the signals were linearly interpolated to obtain values at 1 min resolution.

### Statistical tests

The average amplitude, phase, midline estimating statistic of rhythm (MESOR) (or average measure) and phase differences of the daily sinusoids of acceleration, vertical acceleration and HR were computed for each participant^14^. Within group phase difference between acceleration, vertical acceleration and HR were tested against zero mean using a Mann–Whitney at a 5% significance level. The two-tailed Wilcoxon signed rank test was used to compare the average amplitude, phase and MESOR pairwise across groups, multiple testing was corrected using the positive false discovery rate (FDR) with a conventional significance level of 5%, as defined in Storey^15^.

The coherence between the measured signals and participant mood was calculated at each frequency for each subject group. The coherences were combined to create a signals, which were tested for statistically significant differences between the groups. At each frequency, pairwise tests were performed between the groups using the two-tailed Wilcoxon rank sum test with a 5% significance level. Permutation testing was applied when calculating *p*-values for the coherence in order to correct for the multiple statistical comparisons. Permutation testing was carried out at each frequency, where the labels were randomly assigned to the participants (keeping the same distribution as the true labels) 1000 times, from which the corrected *p*-value is calculated.

## Results

### Diurnal rhythms in objective measures of physiology

The signal of vertical acceleration will be referred to as measuring ‘sleep’, recognising that it is an imperfect estimate of just one element of sleep.

**Fig. 2:**
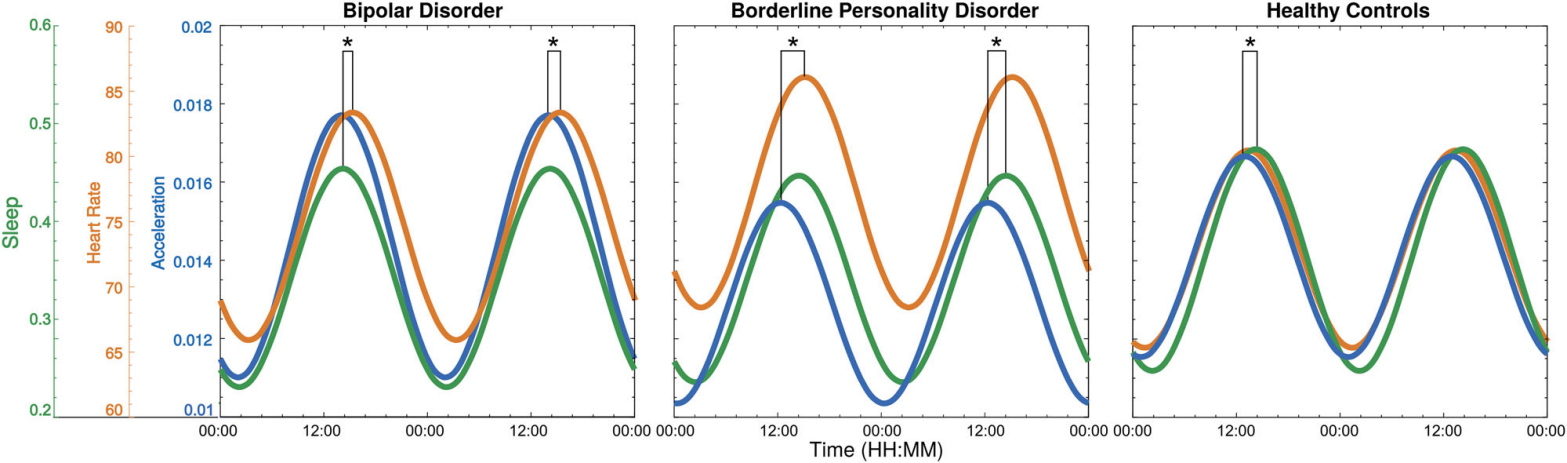
Average sinusoids of sleep, HR and activity shown for two days for each subject. Statistically significant differences (*p* < 0.05) between the phase of each pair of sinusoids, testing if the mean differs from zero, are marked with an asterisk. A significant difference suggests there is a phase lag between the two measures

The average first harmonic sinusoidal fits for the diurnal measures are shown in Fig. 2 for each subject group. For the HC, data largely overlap, with a small phase lag (1.5 h) between activity and sleep, as would be expected: HR is in phase with activity. In BD, the difference between activity and sleep was absent; instead there was significant phase delays in the HR signal of about 1 h compared with sleep and activity. For BPD there was a 2 h phase lag between activity and sleep and almost 3 h delay between peak activity and peak HR. In addition the average HR level in BPD was substantially higher then HC, with BD intermediate, even with similar activity levels across all groups. These statistical differences between groups and within groups are quantified in Table 1. Also in Table 1, the average phase, MESOR and amplitude is compared for activity, sleep and HR across groups; the average values are broadly similar across groups, with the exception of the HR MESOR which was elevated in BPD compared with HC.

### Diurnal rhythms in subjective measures of mood

**Fig. 3:**
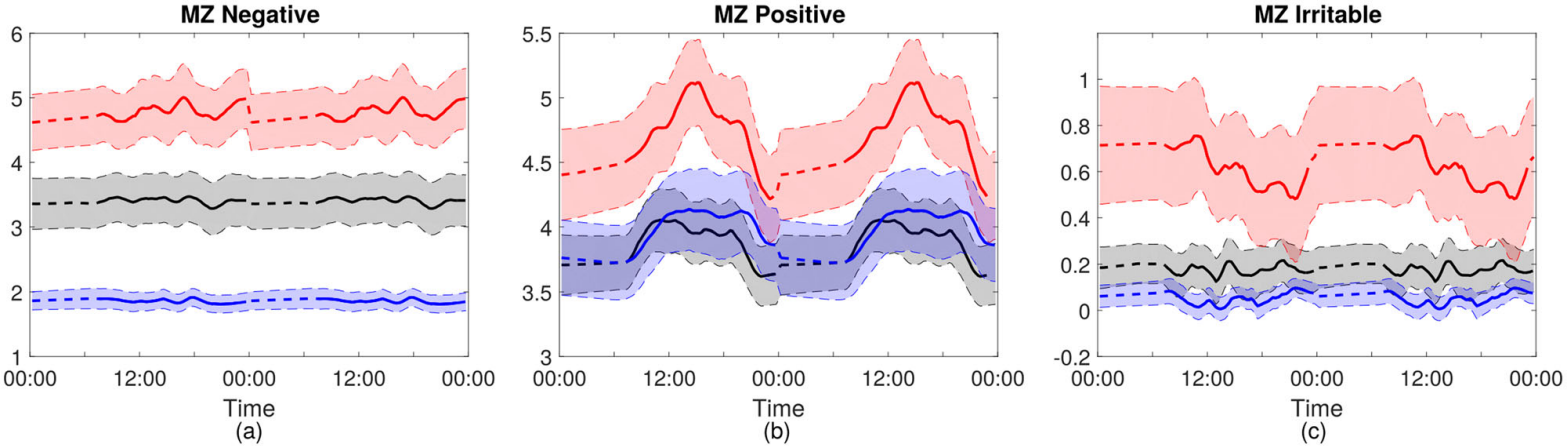
Diurnal patterns (over two days) for each subject group of the average score for the first three principal components of MZ, **a** negative, **b** positive and **c** irritable, repeated over a 48 h period. Solid line represents the mean score, with the dashed period representing the times where no MZ recordings are made. Shaded regions show the standard deviation divided by the square root of group size

Figure 3 shows the mean diurnal patterns of the first three components of MZ for each subject group (shaded region represents±standard error). Negative mood was clearly higher in the patient groups than in controls (Fig. 2a, BPD>BD>HC); cyclicity was not markedly diurnal in any group, although higher ultradian frequencies appeared to occur in BPD. For positive mood, lower frequency quasi-diurnal changes were evident for all groups. Finally, for irritable mood there appeared to be higher frequency ultradian components for all three groups along with different mean levels as for negative mood.

### Effects of medication

Many BD and BPD participants were taking medication which included lithium, antipsychotics, antidepressants, anticonvulsants, anxiolytics and hypnotics. There was no consistent pattern of effect of any drug on any of the measures (see supplementary data).

### The correlation between objective and subjective diurnal measures

As there were either or both diurnal and ultradian variations in mood, especially in the patient groups, an exploratory analysis of the coherence between subjective mood and objective measures of physiology was made across relevant frequencies.

**Fig. 4:**
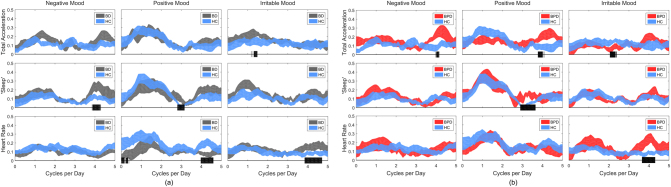
Coherence between total acceleration, sleep and HR signals with negative, positive and irritable MZ scores for: **a** BD and HC participants, and **b** BPD and HC participants. The shaded black bars indicate frequencies at which statistically significant differences between groups were found

At low frequencies (around 1 cycle per 24 h), mood measures were similarly correlated for all three groups. Negative mood and irritable mood was quite weakly associated with physiology, whereas there was generally a greater signal in the association between positive mood and total activity, sleep signal and HR (see Fig. 4),

At higher frequencies (around 4 cycles/24 h), differences were apparent between groups. Negative mood was more highly correlated with activity in both BD and BPD compared with HC. Irritable mood was more highly correlated with HR in both BD and BPD compared to HC.

## Discussion

The total acceleration, vertical acceleration (sleep) and HR signals were strongly diurnal, as expected. HC showed a 1.5 h lag between activity and sleep and HR was in phase with activity. BD showed no lag between activity and sleep but a 1 h phase delay in the HR signal compared with both the other diurnal measures. For BPD there was a 2 h delay between activity and sleep and almost 3 h between activity and HR. The average HR in BPD and BD was higher than HC, but may be explained by antidepressant medication. Negative mood and irritability tended to be correlated with higher frequency components of mood in BD and BPD (at a 5–6 h period) rather than the diurnal cycle. Positive mood correlated with activity and other diurnal measures in all groups. Thus, there appears to be significant de-synchronization between different measures of physiology in BPD and to a lesser extent BD, notably between HR and activity, which are synchronised in HC. The findings suggest that there is an important link between mood and cardiovascular function in BD but particularly in BPD. Both conditions are associated with early cardiovascular mortality and our findings suggest that enhanced mood regulation may be an important factor in reducing cardiovascular risk.

The abnormalities in BD were modest in contrast with the widely held view that BD is, in some quite fundamental sense, a disorder of diurnal function. In fact, the strongest evidence has hitherto related to variability in diurnal function rather than formal evidence for phase changes or de-synchronization. Thus actigraphy studies have shown less stable and more variable diurnal activity in BD even in euthymia^16–19^, with additional disruptions to sleep patterns^20^. In the present study, there was 1 h phase delay for HR in relation to activity, and only trend effects on the level of activity. Increased levels of nocturnal activity have been described in unmedicated young people with hypomanic experience^21^. The normality described here may in part reflect the impact of drug treatment. However, it is difficult to argue without measurement of melatonin or other diurnal variables that diurnal rhythm per se remains a problem or a potential target for management in such patients. Variability in day to day in sleep may be a different matter. A separate analysis has suggested that such variability correlates with mood instability: it is particularly relevant in BD for the sleep cycle and for BPD in the sleep and HR cycles^11^.

BPD is not usually described as being associated with diurnal disturbance, so the effects described here are of great potential interest and clinical importance. Abnormalities of the sleep wake cycle have been described before in patient samples including BPD. Over 20 years ago, a sample of 50 patients selected on the basis of showing delayed sleep phase (43 patients) or free running disorder (7 patients), having consulted for a sleep problem, were described also to show evidence of personality disorder or, at least, psychopathology. There was no particular emphasis on borderline traits and hence the results were highly non-specific. Much more recently, a study in adolescents suggested increased social jetlag in BPD who slept an hour longer on average than matched controls. This is again compatible with significant delayed sleep phase^22^. The delay in estimated sleep phase in BPD implies increased sleep onset latency. Clarification of the prevalence of circadian rhythm sleep disorders (CRSD) in BPD merits further study. It is striking but perhaps not surprising that no emphasis is placed on possible CRSD in the contemporary management of BPD. CRSD has been studied in small populations^23^, but not systematically in large numbers of patients, with phototherapy or drugs like melatonin or agomelatine to phase advance (and so reset) the diurnal cycle. Clinical experience suggests this is highly effective^23^. In a pilot study of a small group of BPD patients with variable sleep/wake profiles, some benefit of light therapy was observed over 3 weeks^24^. Previous sleep investigations in BPD have used polysomnography, with a view to comparing BPD with mood disorder. Thus, a recent systematic review of the relevant literature highlighted the reduced sleep quality in BPD compared with healthy samples and the overlap with reported abnormalities in patients with mood disorder^8^. It correctly identified sleep disorder as a broadly neglected element of the management in BPD.

### Does mood have an important diurnal component?

Diurnal mood variation is an important feature of severe depression^25^, but has received limited attention in studies of euthymic BD and BPD patients. We have shown here that diurnal rhythms of mood exist across participant groups. The use of a simple smart-phone based questionnaire, allowed an uncomplicated method of monitoring mood. Inspection of the average mood profile suggested that positive mood had a significant diurnal component: it was coherent with all three biological measures of diurnal function at the 1 cycle/day frequency in BD and HC groups. BPD showed a weaker relationship with total activity at 1 cycle per day and some activity at a 6 h cycle most seen in HC and BD.

Negative and irritable mood were less coherent with the 1 cycle per day rhythm than positive mood in all participant groups. Negative mood was weakly coherent with a 6 h cycle in BD for sleep and perhaps total activity and in BPD with total activity. Irritable mood was weakly coherent with the 6 h cycle for HR in BD and BPD. The meaning of this 6 h cycle is uncertain, but appears to capture some amplification of mood effects in the evening in the patient groups.

### Limitations

We acknowledge that the cohort in the study is relatively small due to the technical limits of the devices used to record data. Accordingly improved devices will provide future opportunities to replicate and extend the present approach. The participant sample had to be very co-operative in wearing the devices and recording subjective data, so are a selected group in that sense.

The choice of appropriate controls in case control studies is always challenging, because there is a risk of recruiting super healthy individuals unrepresentative of the general population. The present study is strengthened by having two clinical groups whose diurnal physiology show differences from each other and by matching for demographic factors as far as possible including weight.

We did not correct for weekdays and weekend recordings, which will have added to the variance of the recordings, and perhaps reduced our capacity to detect cross-sectional differences in the diurnal behaviour of the groups. Consequently we have emphasised within group desynchronization as our major finding. It is also possible that environmental and behavioural differences existed between the groups, such as timings and composition of food, light exposure, social stress and other factors. These may have found expression in the measures we could make. Finally, most patients were medicated, but with differences between the BD and BPD groups.

The diurnal rhythms of HR variability is a further measure which may exhibit difference between participant groups. The duration of ECG recordings from the participants in this study were not sufficient for analysis of diurnal rhythms of HR variability and the sampling frequency of the HR recorded from the Proteus patch is too low to measure diurnal patterns. Further work will focus on calculating proxies for HR variability from the Proteus patch data and methods for diurnal analysis.

These limitations have to be viewed from the perspective of our primary objective, which was to measure diurnal features between episodes in BD (between crises in BPD). Time between episodes represents an at risk state to which the lived environment contributes, whether on medication or not. The point of intensive subjective or objective monitoring is to identify relevant differences that may serve as a future focus for disease management.

## Conclusions

The phase of diurnal variation in HR is delayed relative to peak activity in BPD (almost 3 h) and BD (1 h), and for sleep 2 h in BPD and not in BD. There abnormalities represent de-synchronization of the diurnal rhythm in HR in BPD and also in BD. Correlations between negative and irritable mood and HR were also increased in the patient groups compared with HC. The findings suggest that regulation of cardiac function is possibly more strongly linked with mood regulation in BPD and BD than in HC. This is interesting in view of the early cardiac mortality associated with both conditions^26^. Diurnal function has been a neglected feature of BPD, which merits as much if not more attention than that accorded to BD. Improved measurement of physiological variables offers important opportunities to refine drug and psychological interventions for hard to treat disorders with marked mood instability.

### Data availability

The datasets analysed during the study are available from the corresponding author on reasonable request. The code used in this study is available at: https://github.com/oliver-carr/Desynchronization-of-Diurnal-Rhythms

### Disclaimer

The views expressed are those of the author(s) and not necessarily those of the NHS, the NIHR or the Department of Health.

## Electronic supplementary material

Supplementary Materials
