# Supplementary material for: Desynchronization of diurnal rhythms in bipolar disorder and borderline personality disorder
Source: Transl Psychiatry. 2018 Apr 12;8:79. doi: 10.1038/s41398-018-0125-7 (PMC5895697; doi:10.1038/s41398-018-0125-7)
Supplement: Supplementary file 1 — Supplementary Materials [file 41398_2018_125_MOESM1_ESM.pdf]

Supplementary Material

A detailed summary of participant information is shown in Table 2. Age, body mass index (BMI), and gender are described, as well as the QIDS score for depression and the Altman score for mania. The table describes participant information for all recruited participants, participants included in each section for analysis, and participants whose data was not analysed.

Table 2. Participant information for: all participants recruited for the study, participants with over four days of acceleration and HR signal used for frequency analysis, participants with over one day of acceleration and HR signal used to find average diurnal rhythms, and participants with no useable days of data who were not used in the analysis.

	Recruited Participants			Participants with signal over four days			Participants with signal over one day			Non-Participants (signals under one day)		
	**BD**	**BPD**	**HC**	**BD**	**BPD**	**HC**	**BD**	**BPD**	**HC**	**BD**	**BPD**	**HC**
**Age (mean±std, years)**	39.0 ±12.9	33.6 ±10.4	36.6 ±13.0	40.9 ±11.4	34.3 ±10.2	43.4 ±14.6	39.7 ±12.6	34.4 ±10.9	40.9 ±13.9	37.4 ±13.9	31.8 ±9.6	29.8 ±8.0
**BMI (mean±std, kg/m²)**	27.3 ±5.1	27.7 ±6.7	23.8 ±3.8	26.1 ±3.9	25.9 ±5.1	24.6 ±4.2	27.2 ±5.1	27.5 ±5.9	24.8 ±4.0	27.4 ±5.3	28.1 ±8.5	22.2 ±2.8
**Male/Female**	20/34	3/28	13/31	4/14	2/12	4/16	13/25	2/20	6/21	7/9	1/8	7/10
**QIDS (mean±std)**	6.94 ±4.86	11.7 ±4.8	1.75 ±1.67	6.65 ±4.53	11.3 ±4.1	1.88 ±1.36	6.72 ±4.97	11.8 ±4.9	1.75 ±1.33	9.33 ±3.06	11.0 ±5.6	1.75 ±2.55
**Altman (mean±std)**	2.72 ±3.22	2.63 ±2.54	0.88 ±2.45	1.65 ±2.00	2.70 ±1.70	0.12 ±0.33	2.61 ±3.08	2.47 ±2.24	0.58 ±1.56	4.00 ±5.19	4.00 ±5.66	1.75 ±4.17

Diurnal Rhythm Quantification

The diurnal analysis was performed on the HR signal, in addition to the integrated total acceleration signal (a measure of total activity) and the integrated vertical acceleration signal (a measure of sleep or rest-activity, due to changing reference angle of the wearer). A single sinusoid was fitted from midday to midday in order to emphasise the night time behaviour, using the model:

$$x_i = M + Acos(\theta_i + \varphi) + e_i \quad (2)$$

where M represents the MESOR (midline estimating statistic of rhythm), A the amplitude and φ the phase shift. e_i represents the error term, assumed to be normally distributed with zero mean and θ_i represents the angle, defined as:

$$\theta_i = \frac{2\pi t_i}{P} \quad (3)$$

where t_i is sample times and P represents the period.

For the case of diurnal rhythms, P is set to 24 hours. This allows the Cosinor equation to be written as:

$$x_i = M + \beta y_i + \gamma z_i + e_i \tag{4}$$

where $\beta = Acos\varphi$, $\gamma = -Asin\varphi$, $y = \cos(2\pi t/P)$ and $z = \sin(2\pi t/P)$. Therefore, eq. 4 is linear in terms of M, β and γ.

Subsequently, a standard ordinary least squares regression approach can be applied to determine the values of M, β and γ which minimise the sum of squares error, $\sum_{i=1}^{N} e_i^2$. The minimum occurs when the first-order derivative of $\sum_{i=1}^{N} e_i^2$ is equal to zero, from which the values of M, β and γ can be found from solving the resulting system of three equations with three unknowns obtained when eq. 5 is squared and differentiated with respect to M, β and γ.

$$\sum_{i=1}^{N} x_i = \sum_{i=1}^{N} M + \beta \sum_{i=1}^{N} y_i + \gamma \sum_{i=1}^{N} z_i + \sum_{i}^{N} e_i \tag{5}$$

This results in a system of equations, where $\omega = 2\pi/P$, shown below:

$$\begin{bmatrix} \sum y_i \\ \sum y_i cos\omega t_i \\ \sum y_i sin\omega t_i \end{bmatrix} = \begin{bmatrix} N & \sum cos\omega t_i & \sum sin\omega t_i \\ \sum cos\omega t_i & \sum cos^2\omega t_i & \sum sin\omega t_i cos\omega t_i \\ \sum sin\omega t_i & \sum sin\omega t_i cos\omega t_i & \sum sin^2\omega t_i \end{bmatrix} \begin{bmatrix} M \\ \beta \\ \gamma \end{bmatrix} \tag{6}$$

where the values of M, β and γ can now be found. To obtain the original parameter values in eq. 2, A and φ must be found. Using the values found for β and γ, it is possible to solve for A:

$$A = (\beta^2 + \gamma^2)^{1/2} \tag{7}$$

and φ:

$$\varphi = \arctan(-\gamma/\beta) \tag{8}$$

Medication Analysis

The Wilcoxon rank sum was performed between all BD and BPD participants combined to test whether medication had a significant effect on each of the diurnal measures. Tables 3, 4 and 5 show the comparison of participants who take and who do not take lithium, antidepressants and antipsychotics respectively. No significant differences were found after correction for multiple tests using the false discovery rate.

Table 3. Comparing average diurnal measures of activity, sleep and heart rate between BD and BPD participants who take lithium and participants who do not. Statistical tests were performed using the Wilcoxon rank sum test, corrected for multiple tests using the false discovery rate at a 5% significance level.

	**With Lithium (n=14)**	**Without Lithium (n=44)**	**FDR**
**Activity**			
**Phase (mean±std)**	5.86e-01±1.04e-01	5.86e-01±1.31e-01	0.791
**MESOR (mean±std)**	1.46e-02±4.50e-03	1.42e-02±3.81e-03	0.793
**Amplitude (mean±std)**	3.19e-03±2.84e-03	3.45e-03±1.83e-03	0.692
**Sleep**			
**Phase (mean±std)**	5.69e-01±5.83e-02	6.04e-01±7.42e-02	0.837
**MESOR (mean±std)**	3.53e-01±7.92e-02	3.35e-01±8.19e-02	0.643
**Amplitude (mean±std)**	1.01e-01±2.77e-02	1.18e-01±1.75e-02	0.532
**Heart Rate**			
**Phase (mean±std)**	6.53e-01±1.22e-01	6.28e-01±8.69e-02	0.710
**MESOR (mean±std)**	7.52e+01±6.73e+00	7.43e+01±8.31e+00	0.627
**Amplitude (mean±std)**	9.01e+00±4.23e+00	8.57e+00±2.31e+00	0.822
**Phase Differences**			
**HR-ACC (mean±std)**	6.68e-02±1.74e-01	4.25e-02±1.37e-01	0.800
**HR-SLP (mean±std)**	8.35e-02±1.41e-01	2.37e-02±6.51e-02	0.756
**ACC-SLP (mean±std)**	1.67e-02±1.03e-01	-1.88e-02±1.35e-01	0.693

Table 4. Comparing average diurnal measures of activity, sleep and heart rate between BD and BPD participants who take antidepressants and participants who do not. Statistical tests were performed using the Wilcoxon rank sum test, corrected for multiple tests using the false discovery rate at a 5% significance level.

	**With Antidepressant (n=29)**	**Without Antidepressant (n=29)**	**FDR**
**Activity**			
**Phase (mean±std)**	5.39e-01±1.45e-01	5.78e-01±1.25e-01	0.697
**MESOR (mean±std)**	1.38e-02±3.87e-03	1.39e-02±3.61e-03	0.742
**Amplitude (mean±std)**	2.84e-03±2.15e-03	3.26e-03±1.79e-03	0.832
**Sleep**			
**Phase (mean±std)**	5.86e-01±7.61e-02	6.06e-01±7.59e-02	0.749
**MESOR (mean±std)**	3.43e-01±7.42e-02	3.41e-01±7.86e-02	0.748
**Amplitude (mean±std)**	1.04e-01±2.80e-02	1.15e-01±1.81e-02	0.584
**Heart Rate**			
**Phase (mean±std)**	6.39e-01±1.15e-01	6.30e-01±8.74e-02	0.726
**MESOR (mean±std)**	7.69e+01±6.33e+00	7.43e+01±7.97e+00	0.698
**Amplitude (mean±std)**	8.94e+00±3.73e+00	8.60e+00±2.70e+00	0.824
**Phase Differences**			
**HR-ACC (mean±std)**	1.00e-01±1.73e-01	5.15e-02±1.28e-01	0.651
**HR-SLP (mean±std)**	5.32e-02±1.18e-01	2.39e-02±7.01e-02	0.635
**ACC-SLP (mean±std)**	-4.68e-02±1.41e-01	-2.76e-02±1.27e-01	0.799

Table 5. Comparing average diurnal measures of activity, sleep and heart rate between BD and BPD participants who take antipsychotics and participants who do not. Statistical tests were performed using the Wilcoxon rank sum test, corrected for multiple tests using the false discovery rate at a 5% significance level.

	**With Antipsychotic (n=27)**	**Without Antipsychotic (n=31)**	**FDR**
**Activity**			
**Phase (mean±std)**	5.73e-01±1.44e-01	5.46e-01±1.29e-01	0.677
**MESOR (mean±std)**	1.34e-02±3.82e-03	1.41e-02±3.64e-03	0.648
**Amplitude (mean±std)**	2.92e-03±2.35e-03	3.17e-03±1.60e-03	0.840
**Sleep**			
**Phase (mean±std)**	5.96e-01±7.46e-02	5.96e-01±7.85e-02	0.732
**MESOR (mean±std)**	3.21e-01±7.98e-02	3.60e-01±6.83e-02	0.541
**Amplitude (mean±std)**	1.11e-01±2.72e-02	1.08e-01±2.14e-02	0.695
**Heart Rate**			
**Phase (mean±std)**	6.37e-01±1.18e-01	6.32e-01±8.59e-02	0.621
**MESOR (mean±std)**	7.59e+01±8.69e+00	7.53e+01±5.84e+00	0.786
**Amplitude (mean±std)**	9.21e+00±3.39e+00	8.39e+00±3.10e+00	0.754
**Phase Differences**			
**HR-ACC (mean±std)**	6.39e-02±1.78e-01	8.61e-02±1.30e-01	0.842
**HR-SLP (mean±std)**	4.11e-02±1.07e-01	3.63e-02±8.99e-02	0.652
**ACC-SLP (mean±std)**	-2.28e-02±1.47e-01	-4.98e-02±1.20e-01	0.954
